# Would 1.0 cm be a more suitable cutoff to subdivide pT1 tumors in hormone receptor‐negative and HER2‐positive breast cancer?

**DOI:** 10.1002/cam4.1785

**Published:** 2018-10-01

**Authors:** Changjun Wang, Yidong Zhou, Hanjiang Zhu, Wei Huang, Ziyuan Chen, Feng Mao, Yan Lin, Xiaohui Zhang, Songjie Shen, Ying Zhong, Yan Li, Qiang Sun

**Affiliations:** ^1^ Department of Breast Surgery Peking Union Medical College Hospital Beijing China; ^2^ Department of Dermatology, 90 Medical Center Way, Surge 110 University of California San Francisco California

**Keywords:** HER2‐enriched breast cancer, hormone receptor, survival, T1 breast cancer

## Abstract

**Background:**

HER2+ and hormone receptor (HoR)‐negative breast cancer usually associated with poor outcome. However, it remained elusive for the prognosis of small (T1a‐T1c) HER2+/HoR‐ breast cancer. The present study retrospectively analyzed the Surveillance, Epidemiology, and End Results (SEER) database to explore the clinicopathological characteristics and prognosis of T1a‐T1c HER2+/HoR‐ breast cancer.

**Material and Methods:**

Data for patients diagnosed with either HER2‐/HoR+or HER2+/HoR‐ T1a‐T1c breast cancer between 2010 and 2012 were obtained from SEER program. Survival analyses were conducted by Kaplan‐Meier method and Cox proportion hazard regression.

**Results:**

Totally, 2648 HER2+/HoR‐ and 56387 HER2‐/HoR+T1a‐T1c breast cancer patients were enrolled. There was a clear trend that tumor size had a positive correlation with advanced AJCC stage (*P* < 0.001) and N‐stage (*P* < 0.001). T1a and T1b HER2+/HoR‐ breast cancer had great homogeneity in that these two subgroups had comparable survival and both showed no significant survival difference with its counterpart of HER2‐/HoR+subtype. Conversely, T1c HER2+/HoR‐ breast cancers revealed worse prognosis than T1a/T1b HER2+/HoR‐ and T1c HER2‐/HoR+tumors (BCSS HR 3.847, *P* < 0.001; OS HR 2.055, *P* < 0.001).

**Conclusion:**

T1a and T1b HER2+/HoR‐ breast cancer had favorable prognosis and great homogeneity, indicating 1.0 cm may be a suitable cutoff for subclassification of T1 cancer. Future randomized clinical trials were warranted to verify this hypothesis and elucidate the biological behavior of small T1 tumor to facilitate precise medicine.

## 1 INTRODUCTION

Breast cancer is one of the most common malignancies in women.^1^ It is presented as a heterogeneous entity with different subtypes characterized with distinctive biological behaviors. HER2‐enriched subtype composed approximately 20% of breast cancer, and HER2 overexpression served as a strong indicator toward poor prognosis even for small‐size T1a, b breast cancer.^2^ The anti‐HER2 monoclonal antibody, trastuzumab, already established its role as the standard regimen for HER2+ breast cancer. Several large‐scale clinical trials proved its efficacy with significant improved survival and reduced recurrence risk up to 50%.^3^, ^4^, ^5^, ^6^

Due to the wide use of screening mammography, there was an increasing trend for pT1a‐pT1b breast cancer (up to 20% of newly diagnosed breast cancer).^7^, ^8^ Despite the dramatic effect of anti‐HER2 agents, no available randomized clinical trials examined the efficacy of adjuvant trastuzumab in pT1a, b HER2+ breast cancers.^9^, ^10^ Besides, the existing retrospective studies reached conflicting results regarding the prognosis of HER2+ T1a, b tumors. The Finnish cancer registry study suggested that pT1a, b N0M0 patients had an excellent prognosis with distant disease‐free survival reached up to 100%.^11^ Whereas, data from a French study revealed HER2 positivity had an independent correlation with cancer recurrence and mortality in anti‐HER2 treatment naïve patients.^12^ These contradictory results made it difficult to balance the survival benefit and potential treatment related toxic effects in managing pT1a‐1c HER2+ cancer.

Additionally, several studies argued whether it was feasible to artificially delineate T1a from T1b and T1c. Rouannet et al^12^ reported the 10‐year prognosis of patients with HER2‐positive tumors was worse than HER2‐negative (disease‐free survival 73% vs 89%), while tumor size (T1a/T1b) was not a relevant prognostic factor. Similarly, another study on Oncotype Dx demonstrated minimal differences in prognosis among these subgroups with almost no impact on cancer‐related deaths.^13^ On the contrary, Fehrenbacher et al^14^ suggested that majority of breast cancer recurrence occurred in tumors measured 1.0 cm, and the recurrence‐free survival was significantly high in T1a tumors compared with 1.0 cm tumors.

Hence, the present study analyzed Surveillance, Epidemiology, and End Results (SEER) database by comparing the survival among T1a‐T1c HER2+/HoR‐ breast cancer to investigate whether tumor size had a dramatic effect on survival for T1 cancers. We also compared survival of T1a‐T1c HER2+ and hormone receptor (HoR)‐negative tumors with HER2‐/HoR+breast cancer to explore the impact of molecular subtype on cancer survival.

## 2 MATERIALS AND METHODS

### 2.1 Patients

The population base data for the study were extracted from the SEER database founded by National Cancer Institute. Relevant case list was generated from SEER 18 incidence database (released April 2016, based on the November 2015 submission).^15^ Detailed clinicopathological information for each case was obtained by SEER*Stat software (version 8.3.2). Details for grouping criteria for breast cancer subtypes by SEER program was presented in Table S1.

Our study was approved by an independent ethical committee/institutional review board Peking Union Medical College Hospital (Peking Union Medical College Ethical Committee) (Protocol No. S‐K516). The data released by the SEER database do not require informed patient consent because cancer is a reportable disease in every state of the United States.

### 2.2 Inclusion and exclusion criteria

The following inclusion criteria were engaged in our study: female patients; diagnosed with breast cancer between 2010 to 2012; age between 18 and 80; breast cancer as the first and only malignant cancer diagnosis; unilateral cancer; pathologically confirmed invasive ductal carcinoma (ICD‐O‐3:8500/3); T1a/T1b/T1c stages; AJCC TNM stages I‐III; histological grade I‐IV; with known ER, PR and HER2 status; breast cancer subtypes were confined to "HER2+/HoR‐" or "HER2‐/HoR+"; and surgical treatment with either mastectomy or breast‐conserving surgery.

The exclusion criteria were listed below: patients diagnosed with breast cancer at death or by autopsy only as well as those with other first primary malignancies or in situ disease; unavailable information about surgery or radiation therapy. Patients diagnosed with breast cancer before 2010 were excluded due to HER2 status was unavailable in SEER database before 2010. Patients diagnosed after 2012 were excluded to ensure adequate follow‐up time that the analysis of 3‐year survival was possible.

### 2.3 Statistical analysis

The demographical and clinicopathological variables as age, race, marital status, laterality, histological grade, AJCC stage, N‐stage, surgery, and radiation therapy were presented as means ± SD and proportions. The statistical significance was assessed by t test for continuous data and Pearson chi‐square or Fisher exact test for categorical data.

Breast cancer‐specific survival (BCSS) was defined as the period between breast cancer diagnosis and death due to breast cancer, while OS referred to the period between breast cancer diagnosis and death due to all causes (including breast cancer). Kaplan‐Meier method was used to plot generate survival curves. Univariate and multivariate survival analyses were conducted by log‐rank test and Cox proportion hazard regression analysis, respectively. Dummy variables were introduced to calculate hazard ratio (HR) for each degree of categorical variables. All the statistical tests were two‐sided, and statistical significance was defined as *P* value <0.05. Statistical analyses were performed under R software (version 3.3.2, R core team, Vienna, Austria, 2016) and its package “Survival” (version 2.40‐1).

## 3 RESULTS

### 3.1 Demographics and clinicopathological characteristics of study population

**Figure 1:**
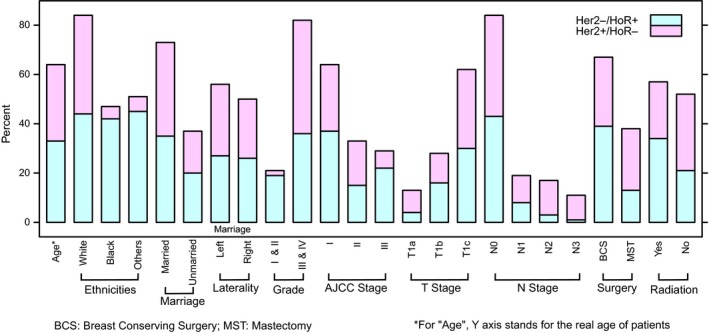
Summary for clinicopathological characteristics of HER2+/HoR‐ and HER2‐/HoR+breast carcinoma

Totally, 2648 HER2+/HoR‐ and 56387 HER2‐/HoR+T1a‐T1c breast cancer patients were enrolled in the present study. Figure 1 summarized the clinicopathological characteristics of HER2+/HoR‐ and HER2‐/HoR+breast carcinoma. In HER2+/HoR‐ breast cancer, there were 481 (18.2%) T1a, 598 (22.6%) T1b, and 1569 (59.2%) T1c tumors. There was a clear trend that tumor size had a positive correlation with advanced AJCC stage (*P* < 0.001) and N‐stage (*P* < 0.001). T1a tumor had a relatively high proportion of Stage I (92.9%) and N0 (90.9%), compared with T1b (Stage I 82.9% and N0 79.6%) and T1c (Stage I 71.9% and N0 66.9%). Although statistical significance existed among T1a/T1b/T1c, there was an overwhelming large part (more than 90%) of HER2+/HoR‐ tumors that had histological grade III/IV. Similarly, despite data showed T1b patients had a younger age than T1c and T1a was prone to affect black people, the absolute difference was neglectable, indicating that all the T1a/T1b/T1c patients developed cancer symptoms in their fifties and more than 70% were Caucasians. There was no significant difference of marital status and laterality among all the three T1 subgroups. Notably, compared to both T1b and T1c, T1a patients received significantly more mastectomy than breast‐conserving surgery (BCS) (mastectomy: T1a 59.1%, T1b 48.2% and T1c 45.2%, *P* < 0.001), while T1b and T1c had a comparable mastectomy rate (*P* = 0.236). As for radiation therapy, less patients with T1a tumor (37.4%) underwent radiotherapy than T1c (45.5%, *P* = 0.002). Comparisons between T1a/T1b/T1c, clinicopathological parameters were provided in Tables 1, 2, and Table S2.

**Table 1:** Clinicopathological characteristics of T1a and T1b Her2+/HoR‐ breast carcinoma

Characteristics	T1a (N = 481)	T1b (N = 598)	*P* value
Median Follow‐up (months) (IQR)	22.0 (9.0‐34.0)	23.0 (11.0‐36.0)	
Age (Mean ±SD)	56.5 ± 10.6	57.7 ± 10.7	0.067
Race			
White	347 (72.4%)	458 (77.2%)	**<0.001** *
Black	40 (8.4%)	70 (11.8%)	
Others[Link]	92 (19.2%)	65 (11.0%)	
Marital status			
Married	337 (73.1%)	377 (66.8%)	0.036
Not married[Link]	124 (26.9%)	187 (33.2%)	
Laterality			
Left	255 (53.0%)	324 (54.2%)	0.749
Right	226 (47.0%)	274 (45.8%)	
Grade			
I	0 (0.0%)	0 (0.0%)	0.400[Link]
II	15 (3.3%)	13 (2.2%)	
III/IV	440 (96.7%)	566 (97.8%)	
AJCC stage			
I	447 (92.9%)	496 (82.9%)	**<0.001** *
II	29 (6.0%)	73 (12.2%)	
III	5 (1.1%)	29 (4.9%)	
N‐Stage			
N0	437 (90.9%)	476 (79.6%)	**<0.001** *
N1	39 (8.1%)	93 (15.5%)	
N2	3 (0.6%)	19 (3.2%)	
N3	2 (0.4%)	10 (1.7%)	
Surgery			
BCS	196 (40.9%)	303 (51.8%)	**<0.001** *
Mastectomy	283 (59.1%)	282 (48.2%)	
Radiation			
Yes	176 (37.4%)	248 (43.5%)	0.052
No	295 (62.6%)	322 (56.5%)	

BCS, breast‐conserving surgery; IQR, interquartile range

Other includes American Indian/Alaskan native, and Asian/Pacific Islander.

Not married includes divorced, separated, single (never married), unmarried or domestic partner and widowed.

Items with zero value were not included for chi‐square test.

a Statistical significance.

**Table 2:** Clinicopathological characteristics of T1a and T1c Her2+/HoR‐ breast carcinoma

Characteristics	T1a (N = 481)	T1c (N = 1569)	*P* value
Median Follow‐up (months)(IQR)	22.0 (9.0‐34.0)	22.0 (10.0‐34.0)	
Age (Mean ±SD)	56.5 ± 10.6	56.3 ± 11.4	0.736
Race			
White	347 (72.4%)	1129 (72.3%)	**<0.001** *
Black	40 (8.4%)	223 (14.3%)	
Others[Link]	92 (19.2%)	210 (13.4%)	
Marital status			
Married	337 (73.1%)	955 (64.6%)	**<0.001** *
Not married[Link]	124 (26.9%)	523 (35.4%)	
Laterality			
Left	255 (53.0%)	795 (50.7%)	0.396
Right	226 (47.0%)	774 (49.3%)	
Grade			
I	0 (0.0%)	0 (0.0%)	**<0.001** * **^,^** [Link]
II	15 (3.3%)	14 (0.9%)	
III/IV	440 (96.7%)	1501 (99.1%)	
AJCC Stage			
I	447 (92.9%)	1128 (71.9%)	**<0.001** *
II	29 (6.0%)	325 (20.7%)	
III	5 (1.1%)	116 (7.4%)	
N‐Stage			
N0	437 (90.9%)	1050 (66.9%)	**<0.001** *
N1	39 (8.1%)	403 (25.7%)	
N2	3 (0.6%)	76 (4.8%)	
N3	2 (0.4%)	40 (2.6%)	
Surgery			
BCS	196 (40.9%)	836 (54.8%)	**<0.001** *
Mastectomy	283 (59.1%)	690 (45.2%)	
Radiation			
Yes	176 (37.4%)	675 (45.5%)	**0.002** *
No	295 (62.6%)	810 (54.5%)	

BCS, breast‐conserving surgery; IQR, interquartile range.

Other includes American Indian/Alaskan native, and Asian/Pacific Islander.

Not married includes divorced, separated, single (never married), unmarried or domestic partner and widowed.

Items with zero value were not included for chi‐square test.

a Statistical significance.

Regarding HER2‐/HoR+breast carcinoma, most of the clinicopathological parameters indicated favorable prognosis compared to HER2+/HoR‐ subtype, including age (*P* < 0.001), histological grade (*P* < 0.001), AJCC stage (*P* < 0.001), and N‐stage (*P* < 0.001). One exception was that HER2+/HoR‐ subtype had more T1a tumor than HER2‐/HoR+ (18.2% vs 11.8%, *P* < 0.001), indicating more aggressive subtype presented with smaller size tumors. Besides, more patients in HER2‐/HoR+group received BCS (*P* < 0.001) and radiation therapy (*P* < 0.001). Detailed information was summarized in Table 3.

**Table 3:** Clinicopathological characteristics of Her2+/HoR‐ and Her2‐/HoR+breast carcinoma

Characteristics	Her2+/HoR‐ (N = 2648)	Her2‐/HoR+ (N = 56387)	*P* value
Median Follow‐up (months)(IQR)	22.0 (10.0‐35.0)	22.0 (10.0‐34.0)	
Age (Mean ±SD)	56.6 ± 11.1	59.9 ± 11.1	**<0.001** *
Race			
White	1934 (73.4%)	46204 (82.5%)	**<0.001** *
Black	333 (12.7%)	4536 (8.1%)	
Others [Link]	367 (13.9%)	5273 (9.4%)	
Marital Status			
Married	1669 (66.7%)	33876 (63.2%)	**<0.001** *
Not Married [Link]	834 (33.3%)	19752 (36.8%)	
Laterality			
Left	1374 (51.9%)	1397 (50.3%)	0.122
Right	1274 (48.1%)	1335 (49.7%)	
Grade			
I	0 (0%)	0 (0%)	**<0.001** * **^,^** [Link]
II	42 (1.6%)	19911 (35.9%)	
III/IV	2507 (98.4%)	35494 (64.1%)	
AJCC Stage			
I	154 (50.3%)	1773 (64.9%)	**<0.001** *
II	109 (35.6%)	851 (31.1%)	
III	43 (14.1%)	109 (4.0%)	
T Stage			
T1a	481 (18.2%)	6640 (11.8%)	**<0.001** *
T1b	598 (22.6%)	17997 (31.9%)	
T1c	1569 (59.2%)	31750 (56.3%)	
N‐Stage			
N0	1963 (74.1%)	46018 (81.6%)	**<0.001** *
N1	535 (20.2%)	9071 (16.1%)	
N2	98 (3.7%)	1001 (1.8%)	
N3	52 (2.0%)	297 (0.5%)	
Surgery			
BCS	1335 (51.5%)	39425 (70.8%)	**<0.001** *
Mastectomy	1255 (48.5%)	16246 (29.2%)	
Radiation			
Yes	1099 (43.5%)	33773 (61.6%)	**<0.001** *
No	1427 (56.6%)	21060 (38.4%)	

BCS, breast‐conserving surgery; IQR, interquartile range.

Other includes American Indian/Alaskan native, and Asian/Pacific Islander.

Not married includes divorced, separated, single (never married), unmarried or domestic partner and widowed.

Items with zero value were not included for chi‐square test.

a Statistical significance.

### 3.2 Survival analysis among T1a/T1b/T1c HER2+/HoR‐ subtype

**Figure 2:**
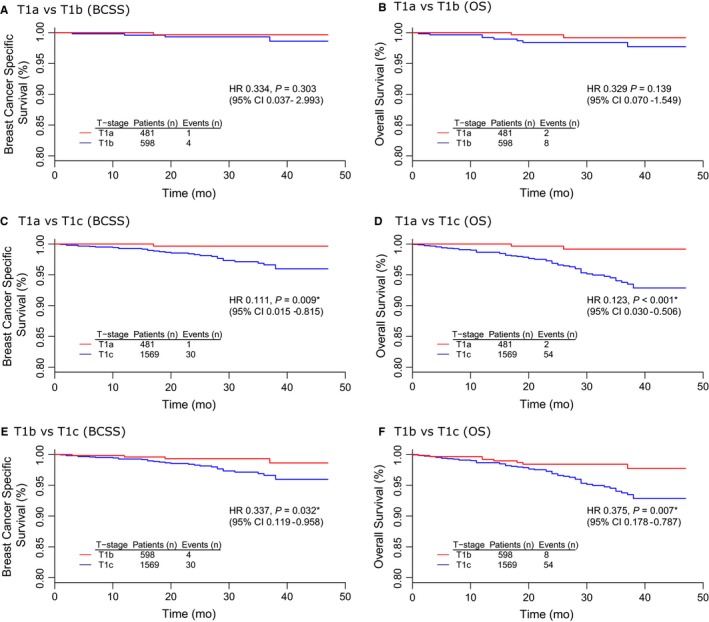
Kaplan‐Meier estimates of breast cancer‐specific survival (BCSS) and overall survival (OS) for T1a/T1b/T1c HER2+/HoR‐ breast cancer: A, T1a vs T1b (BCSS); B, T1a vs T1b (OS); C, T1a vs T1c (BCSS); D, T1a vs T1c (OS); E, T1b vs T1c (BCSS); F, T1b vs T1c (OS)

Table 4 summarized the results of univariate and multivariate survival analyses among T1a/T1b/T1c HER2+/HoR‐ tumors. The corresponding survival curves were presented in Figure 2. There was no significant difference between T1a and T1b survival in terms of BCSS (univariate *P* = 0.303, multivariate *P* = 0.850) and overall survival (OS) (univariate *P* = 0.139, multivariate *P* = 0.999) (Figure 2A,B). In contrast, T1a had a better prognosis than T1c with prolonged BCSS (HR 0.111, 95% CI 0.015‐0.815, *P* = 0.009) and OS (HR 0.123, 95% CI 0.030‐0.506, *P* < 0.001) by univariate analysis (Figure 2C,D). Moreover, the comparison between T1b and T1c also proved that T1b had a dramatic survival advantage over T1c in terms of BCSS (univariate: HR 0.337, 95% CI 0.119‐0.958, *P* = 0.032; multivariate: HR 0.325, 95% CI 0.113‐0.928, *P* = 0.036) and OS (univariate: HR 0.375, 95% CI 0.178‐0.787, *P* = 0.007; multivariate: HR 0.364, 95% CI 0.172‐0.770, *P* = 0.008) (Figure 2E,F).

**Table 4:** Survival analyses of BCSS and OS among T1a, T1b and T1c stage of Her2+/HoR‐ breast carcinoma

	Univariate		Multivariate	
	Hazard ratio (95% CI)	*P* value	Hazard ratio (95% CI)	*P* value
T1a vs T1b				
BCSS	0.334 (0.037‐2.993)	0.303	0.626 (0.005‐70.91)	0.850
OS	0.329 (0.070‐1.549)	0.139	NS	0.999
T1a vs T1c				
BCSS	0.111 (0.015‐0.815)	**0.009** *	NS	0.998
OS	0.123 (0.030‐0.506)	**<0.001** *	NS	0.998
T1b vs T1c				
BCSS	0.337 (0.119‐0.958)	**0.032** *	0.325 (0.113‐0.928)	**0.036** *
OS	0.375 (0.178‐0.787)	**0.007** *	0.364 (0.172‐0.770)	**0.008** *

CI, confidence interval; NS, Non‐significant.

a Statistical significance.

### 3.3 Survival analyses between HER2+/HoR‐ and HER2‐/HoR+subtypes

**Figure 3:**
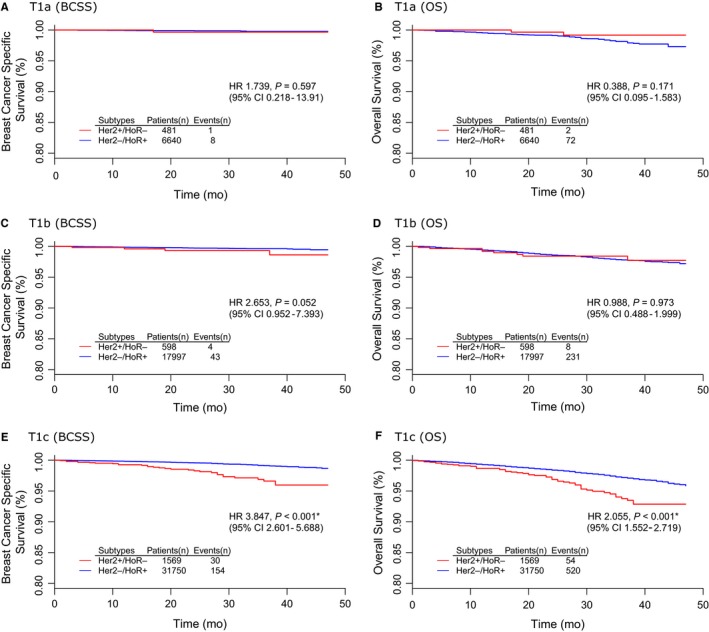
Survival analyses between HER2+/HoR‐ and HER2‐/HoR+breast cancers in different T1 subgroups: A, T1a (BCSS); B, T1a (OS); C, T1b (BCSS); D, T1b (OS); E, T1c (BCSS); F, T1c (OS)

Table 5 summarized the results of univariate and multivariate survival analyses between HER2+/HoR‐ and HER2‐/HoR+subtypes. The corresponding survival curves were presented in Figure 3. There were no significant BCSS or OS differences between HER2+/HoR‐ and HER2‐/HoR+subtypes either in T1a (Figure 3A,B) or T1b group (Figure 3C,D). It was of notice that the survival difference reached statistical significance only in T1c group. HER2+/HoR‐ T1c tumors had both poorer BCSS (HR 3.847, 95% CI 2.601‐5.688, *P* < 0.001) and OS (HR 2.055 95% CI 1.552‐2.719, *P* < 0.001) than its counterparts of HER2‐/HoR+subtype (Figure 3E,F).

**Table 5:** Survival analyses of BCSS and OS between Her2+/HoR‐ and Her2‐/HoR+subtypes in T1a, T1b and T1c stage breast carcinoma

	Univariate		Multivariate	
	Hazard ratio (95% CI)	*P* value	Hazard ratio (95% CI)	*P* value
T1a (Her2+/HoR‐ vs Her2‐/HoR+)				
BCSS	1.739 (0.218‐13.91)	0.597	NS	1.000
OS	0.388 (0.095‐1.583)	0.171	NS	0.999
T1b (Her2+/HoR‐ vs Her2‐/HoR+)				
BCSS	2.653 (0.952‐7.393)	0.052	0.584 (0.066‐5.151)	0.628
OS	0.988 (0.488‐1.999)	0.973	0.738 (0.163‐3.343)	0.694
T1c (Her2+/HoR‐ vs Her2‐/HoR+)				
BCSS	3.847 (2.601‐5.688)	<0.001*	0.835 (0.281‐2.484)	0.746
OS	2.055 (1.552‐2.719)	<0.001*	0.784 (0.367‐1.678)	0.531

CI, confidence interval; NS, Non‐significant.

a Statistical significance.

## 4 DISCUSSION

The present study investigated 2648 HER2+/HoR‐ and 56387 HER2‐/HoR+T1a‐T1c breast cancer patients. Regarding to survival analyses within HER2+/HoR‐ subtype, T1a had similar outcome with T1b tumor, while both T1a and T1b had significant survival advantage over T1c in terms of BCSS and OS. Additionally, the comparison between HER2+/HoR‐ and HER2‐/HoR+subtypes also revealed that survival difference only existed when tumors size reached T1c stage, neither T1a or T1b subgroup had prominent impact on BCSS or OS in these two molecular subtypes. Through this retrospective study, it can be speculated that patients with T1a and T1b diseases had a strong homogeneity that a cutoff value with 1 cm to subdivide T1 may be a more practical module to facilitate clinical decision making.

Generally, small tumor size was regarded as a favorable prognostic indicator for breast cancer. This was in line with our finding that T1a accompanied with low AJCC stage, N‐stage, and high histological grade. On the contrary, our data also showed T1a accounted for a greater proportion of aggressive molecular subtype, such as HER2+/HoR‐ (18.2%), compared with HER2‐/HoR+ (11.8%). Correspondingly, patients with T1a HER2+/HoR‐ disease received more aggressive surgical treatment (mastectomy, 48.5%) than HER2/HoR‐ (49.2%). It raised the concern that traditional favorable clinicopathological parameters (such as tumor size and N‐stage) were not necessarily correlated to good prognosis, while the tumor intrinsic characteristics (like molecular subtype) may play a dominant role in cancer biological behavior. Breast cancer intrinsic subtypes was first introduced by Perou et al in 2000 who analyzed 65 cancer tissue samples by gene microarray and divided breast cancer into five subtypes—Luminal A, Luminal B, HER2‐enriched, triple‐negative, and normal‐like.^16^ HER2‐enriched subtype had a great overlap with immunohistochemical profile HER2+/HoR‐, and HER2 positivity was a potent indicator for poor prognosis.^17^ HER2+/HoR‐ subtype usually had a lower incidence for T1a tumor than advanced T stage.^18^ Study by Lannin et al also noticed that many small tumors with favorable biologic features do not progress to large tumor, and to some extent, tumor size may just be a proxy for favorable or unfavorable tumor biologic features.^19^ And this rationale could also partially explain the phenomenon that interval breast cancer had worse prognosis than screen‐detected small tumors.^20^, ^21^

Given the favorable outcome of T1a and T1b breast cancer, these small cancers were excluded from large clinical trials.^18^ Thus, the prognostic information was scare and the optimal treatment for small HER2‐enriched breast cancer remained undetermined. Although NCCN guideline recommended chemotherapy with anti‐HER2 agent for all T1a‐T1c patients, it also claimed that the prognosis of this patient group remained elusive.^22^ The present study retrospectively analyzed the BCSS and OS for HER2+/HoR‐ T1a‐T1c breast cancer and proved that T1a had similar outcome with T1b, and both T1a and T1b were superior to T1c, which implied 1.0 cm may be a better option for T1 subclassification. This concept was also supported by the comparison with HER2‐/HoR+cancers that only T1c tumor showed survival advantage of HER2‐/HoR+subtype. Our results were concordant with the study by Vaz‐Luis et al that enrolled 4113 T1a, b patients with 520 HER2+ breast cancer and demonstrated that these untreated study population had a 5 years OS over 95%.^23^ And in several retrospective studies, HER2 status was not confirmed as an independent prognostic indicator for survival in small HER2+ breast cancer.^23^, ^24^ Moreover, study by Ramshorst et al provided additional evidence that only T1c benefited from systemic treatment and survival difference between treated and untreated T1a‐1b tumors was neglectable.^25^ In contrast, several studies indicated small tumors with worse outcome. Without the confounding effect of trastusumab, a cohort of 98 HER2+ T1a, b breast cancers with no adjuvant treatment showed significant worse disease‐free survival and distant disease‐free survival compared to HER2‐ subgroup.^26^ Another study revealed small (<1 cm) HER2+ breast cancer has a high risk for recurrence with HR up to 8.8.^27^

HER2+ had long been considered as an indicator for poor outcome. Before the invention of trastusumab, HER2+ breast cancer was considered to have the worst prognosis among all the subtypes. Consequently, HER2+ breast cancer usually received intensive treatment with concurrent chemo‐ and anti‐HER2 therapy. This notion also influenced the surgical options in that our data exhibited larger portion of HER2+/HoR‐ T1 patients underwent mastectomy, rather than BCS. However, in the era of numerous available anti‐HER2 agents, such concerns about disease recurrence were largely unnecessary. In the recent published APHINITY trial, the dual anti‐HER2 with trastusumab and pertusumab could improve 5‐year OS to near 98%.^28^ Another trial also proved taxanes with trastusumab could be an optimal option for small HER2+ tumor with 3‐year rate of survival free from invasive disease was 98.7%.^29^ All the above information implied a de‐escalating strategy for the management of T1a and T1b HER2+ breast cancer. In addition, although treatment data were absent for SEER database, it can be speculated that less T1a patients received anti‐HER2 treatment than T1b and T1c. This potential bias also favored our result in that T1a received less intensive treatment and achieved comparable efficacy as T1b. Considering anti‐HER2 therapy usually combined with chemotherapy, further clinical trials should focus on less intensive therapy, such as anti‐HER2 therapy alone or combined with endocrine therapy, to deliver personalized medicine.

The present study had several limitations. First, since the SEER database did not include any treatment information regarding anti‐HER2 therapy, the efficacy of trastuzumab on small HER2+/HoR‐ cancers was unable to evaluate. And given the dramatic antineoplastic effect of trastuzumab, its application in T1a tumors could potentially mask the impact of tumor size and the other clinicopathological parameters. Second, due to the retrospective nature of the present study, the unbalanced baseline characteristics, especially for adjuvant systemic treatment, could potentially introduce bias to survival analyses. Although multivariate statistical approaches were engaged to account for cohort differences, it may not thoroughly eliminate the selection bias in study population. Thus, the present data were largely descriptive and confirmative conclusions were difficult to draw.

In conclusion, our study analyzed SEER database to summarize the clinicopathological features of T1a‐T1c HER2+/HoR‐ breast cancer. T1a and T1b tumors shared great similarity in terms of BCSS and OS, while T1c tumors had a significant poor prognosis. Besides, T1a and T1b HER2+/HoR‐ also presented as a comparable outcome with its counterpart of HER2‐/HoR+subtypes. The above results proposed the hypothesis that 1.0 cm may be a more suitable cutoff to define T1 subclasses for HER2+/HoR‐ tumors. Future prospective studies are warranted to investigate the biological behaviors of small breast cancer to tailor personalized medicine.

## 5 CONCLUSION

T1a and T1b HER2+/HoR‐ breast cancer had great homogeneity and favorable prognosis, indicating 1.0 cm may be a suitable cutoff for subclassification of T1 cancer. Future large‐scale randomized clinical trials were warranted to verify this hypothesis and elucidate the biological behavior of small T1 tumor to facilitate precise medicine.

## CONFLICT OF INTEREST

None declared.
