# Supplementary material for: Would 1.0 cm be a more suitable cutoff to subdivide pT1 tumors in hormone receptor‐negative and HER2‐positive breast cancer?
Source: Cancer Med. 2018 Oct 1;7(11):5420–30. doi: 10.1002/cam4.1785 (PMC6246936; doi:10.1002/cam4.1785)
Supplement: Supplementary file 1 [file CAM4-7-5420-s001.docx]

**Supplement Table 1: SEER Variable and Recode definition for “Breast Subtype (2010+) ”**

| **“Breast Subtype (2010+)”**  **Value** | **Year** | **Derived HER2 Recode (2010+)** | **ER / PR Status Recode Breast Cancer (1990+)** |
| --- | --- | --- | --- |
| **“Not 2010+ Breast”** | Before 2010 |  |  |
| **“HER2+/HoR+”** | After 2010 | Positive | ER/PR = positive / borderline* |
| **“HER2+/HoR-”** | After 2010 | Positive | Both are negative |
| **“HER2-/HoR+”** | After 2010 | Negative | ER/PR = positive / borderline* |
| **“Triple Negative”** | After 2010 | Negative | Both are negative |
| **“Unknown”** | All other values | | |

*In data submissions earlier than November 2014, borderline ER/PR was not classified with positive. These cases (n=120 in the November 2013 data) would have been unknown.

Please refer the following website for details about the definition of “Breast Subtype (2010+)” in SEER database:

<https://seer.cancer.gov/seerstat/databases/ssf/breast-subtype.html>

**Supplement Table 2: Clinicopathological characteristics of T1b and T1c Her2+/HoR- breast carcinoma**

| **Characteristics** | | **T1b**  **(N = 598)** | **T1c**  **(N = 1569)** | ***p* value** |
| --- | --- | --- | --- | --- |
| **Median Follow-up (months)(IQR)** | | 23.0 (11.0-36.0) | 22.0 (10.0-34.0) |  |
| **Age (Mean ± SD)** | | 57.7 ± 10.7 | 56.3 ± 11.4 | **0.013*** |
| **Race** | **White** | 458 (77.2%) | 1129 (72.3%) | 0.066 |
|  | **Black** | 70 (11.8%) | 223 (14.3%) |  |
|  | **Others ^a^** | 65 (11.0%) | 210 (13.4%) |  |
| **Marital Status** | **Married** | 377(66.8%) | 955 (64.6%) | 0.371 |
|  | **Not Married ^b^** | 187 (33.2%) | 523 (35.4%) |  |
| **Laterality** | **Left** | 324 (54.2%) | 795 (50.7%) | 0.157 |
|  | **Right** | 274 (45.8%) | 774 (49.3%) |  |
| **Grade** | **I** | 0 (0.0%) | 0 (0.0%) | **0.029*^c^** |
|  | **II** | 13 (2.2%) | 14 (0.9%) |  |
|  | **III / IV** | 566 (97.8%) | 1501 (99.1%) |  |
| **AJCC Stage** | **I** | 496 (82.9%) | 1128 (71.9%) | **< 0.001*** |
|  | **II** | 73 (12.2%) | 325 (20.7%) |  |
|  | **III** | 29 (4.9%) | 116 (7.4%) |  |
| **N Stage** | **N0** | 476 (79.6%) | 1050 (66.9%) | **< 0.001*** |
|  | **N1** | 93 (15.5%) | 403 (25.7%) |  |
|  | **N2** | 19 (3.2%) | 76 (4.8%) |  |
|  | **N3** | 10 (1.7%) | 40 (2.6%) |  |
| **Surgery** | **BCS** | 303 (51.8%) | 836 (54.8%) | 0.236 |
|  | **Mastectomy** | 282 (48.2%) | 690 (45.2%) |  |
| **Radiation** | **Yes** | 248 (43.5 %) | 675 (45.5 %) | 0.457 |
|  | **No** | 322 (56.5%) | 810 (54.5%) |  |

**Abbreviation**: BCS: breast conserving surgery; IQR: interquartile range.

* indicates statistical significance.

^a^ Other includes American Indian/Alaskan native, and Asian/Pacific Islander.

^b^ Not married includes divorced, separated, single (never married), unmarried or domestic partner and widowed.

^c^ Items with zero value were not included for Chi-square test.
